# The Use of the Obstetrical Forceps*Read before the Fifth and Sixth District Medical Society, Corpus Christi, Texas, September 7-8, 1914.

**Published:** 1915-02

**Authors:** T. J. Turpin

**Affiliations:** Corpus Christi, Texas


### The Use of the Obstetrical Forceps.*

BY T. J. TURPIN, M. D., CORPUS CHRISTI, TEXAS.

In the course of conversation with a friend a few days ago, he said he had noticed that I and other doctors were to read papers before this society, and he asked why, since one could get all there was upon any subject from "The Text-Books."

I was rather puzzled to answer him, for I could give him no good reason for such papers as we are supposed to read, unless it is that we are expected to write of our experiences rather than of a rehash of "The Text-Books."

With this idea in view, I shall not take up the well known and almost self-evident propositions of my subject, but will confine my remarks to such points as have seemed to me the most important in a practice of forty-five years.

My subject is "The Use of the Obstetrical Forceps."

Madam Baudeloque, one of the most famous obstetricians of earlier days, asserted that "The forceps were the most useful of all surgical instruments," and there are many of the present day who think her assertion not an exaggeration.

Offering, as they do, artificial hands which may be applied to the fetal head without injury, they make possible that which the unaided hands cannot accomplish.

There is a general prejudice among the laity against the forceps, as they are supposed to be the cause of perineal lacerations. I know some doctors who tell their patients that forceps are the cause of such accidents.

---

*Read before the Fifth and Sixth District Medical Society, Corpus Christi, Texas, September 7-8, 1914.

I believe this is a mistake; for if the forceps are properly used, taking time enough to allow the perineum to dilate—holding back the head when the pains are strong—I am sure that, instead of causing rupture, they will often prevent it.

The forceps do not increase the diameter of the head. Upon the contrary, it is possible to compress the head with them nearly half in inch with safety. There is, therefore, no reason for believing that they increase the danger of perineal rupture, except that they make rapid delivery more possible before the perineum has had time to dilate.

Most of us are apt to apply too much force and to be in too great a hurry with forceps, and this is especially true when the husband and mother are wringing their hands and asking if the patient has had too much chloroform, or if she is not dying.

I have said nothing about contracted pelvis, nor abnormally large heads, for the reason that I know very little about such conditions. In a practice of forty-five years, with about twenty-five hundred deliveries, I have met with only one deformed pelvis. This was a twin case, and the first child came breech foremost, but the head caught, on the brim of the pelvis, and was delivered with forceps.

Three kinds of forceps have been in general use in the United States—the short straight, the axistraction of De Tarnier and the long double curved forceps. The short forceps are only suitable for cases when the head is low down in the hollow of the sacrum; and the long forceps may be substituted in all cases. The De Tarnier forceps are of especial service in high operations, when the head has not engaged in the superior strait. Traction with the De Tarnier forceps is more direct, but the force applied is harder to estimate, and I think there is more danger of injury to the child's head in the use of them than with other forceps.

Theoretically, the traction forceps should be the best, but I have never found great difficulty in applying the long forceps, perhaps because I am much more accustomed to them.

When the long forceps have been applied above the brim of the pelvis I have sometimes found that a piece of bandage through the feuestra of the blades will make the traction more direct, and will accomplish all that the axis instrument can do.

Forceps should not be used to save the time of the doctor. This rule will, no doubt, be approved by most of us; but I venture to say that some of us have violated it at least once.

The perineum ruptures in a certain proportion of cases whether one uses forceps or not.

Forceps should never be used until the patient is completely anesthetized.

Forceps should not be used until a thorough examination has been made with the whole hand, not the fingers. The hand can go anywhere the forceps can.

Forceps should not be used until there is complete dilatation of the os uteri, "except in cases of great urgency."

Forceps *should*, as a rule, not be applied above the brim of the pelvis, unless there is urgent need of prompt delivery.

Forceps *should* be used whenever there is danger to the life of the mother or child from delay.

In eclampsia, all authorities agree that no matter what the time of pregnancy, no matter whether the os is dilated or not, the uterus must be emptied as soon as possible. Lacerations of the os or perineum are trivial compared with the danger that threatens the life of the patient. To make prompt delivery, the os must first be dilated enough to allow the introduction of the hand to make out the presentation.

If the head has not engaged, and is still above the brim of the pelvis, it is often a question whether to apply forceps or to turn and deliver by the feet.

I believe where the head has not engaged, and there is urgent need of haste, that it is easier and better to turn and deliver by the feet. In such case it is better to apply the forceps to the after-coming head in order to expedite the delivery. This also applies to hemorrhage from placenta previa, for the body of the child generally makes a better plug to stop hemorrhage than the head does.

There is also an urgent need of prompt delivery when there is a prolapsed cord which cannot be replaced. As far as my experience goes, prompt delivery is the only thing to do with a prolapsed cord, for I have never been able to replace one.

Forceps *should* be used when the labor has been prolonged, to save the mother from suffering. This proposition will be disputed by many doctors who insist that "meddlesome midwifery" is bad, and that "nature should be allowed to take her course."

Most women can bear six or eight hours' hard labor pains—the weaker ones are often those who bear pain best.

We know that we can use forceps so as to shorten labor. We know that, in the hands of one who knows his business, the forceps will do no harm to the mother, and no serious injury to the child. Why, then, should we let the woman suffer twenty-four or more hours when we can relieve her? We give chloroform or ether to avoid pain of surgical operations, when there is always a certain risk. Why not do the same for a woman, when satisfied that there is no danger? I think it much better to apply forceps as soon as the perineum is dilated than to wait eighteen or twenty hours, even if one gives chloroform freely to relieve pain; and I feel sure that forceps delivery is infinitely safer than the "Twilight Sleep," so much talked of lately.

---

Fort Worth, Texas, December 7, 1914.

*Mrs. F. E. Daniel, Austin, Texas.*

Dear Mrs. Daniel: Your request for something about pellagra has been received. Don't know just what you want to know about it so will go over what seem to me at this time to be some of the most important features of the disease.

As to the cause of pellagra we are almost as much in the dark now as we were when it first came to us, when the Italians told us it was due to eating corn products that were in some way diseased. However, most of the American physicians do not agree with that theory now, though there are a few who still cling to it for want of a better one. Others have been responsible for new theories, some of which seem based on good reasoning, while others seem to have nothing but thin air to support them. Possibly the most widely accepted theory among the American physicians today is that it is a protozoan disease, transmitted by an insect; and that, most likely, the stable fly, Stomoxys Calcitrans.

It is the history of all insect born diseases (in the sense that malaria is an insect born disease) that they are carried from the infected patient to the uninfected patient by the insect's first biting the infected patient and thus getting the blood in its stomach, where the germ passes through a separate cycle of existence, then biting the uninfected patient and discharging the new-form germ into the tissues through the saliva. Such diseases occur mainly in the temperate and tropical zones, where the warm season lasts longer and the insect thus has a longer period of existence; and while they also occur in colder climates, it is for a shorter period, and the diseases are less prevalent there. Thus it is with malaria now, and was with yellow fever when we had it in this country. And so it seems to me that we can account in this way for the present pellagra situation, that is, lots of it in the South and not so much in the North.

Then, too, in the South the disease appears mainly in the spring and early summer, while in the North many cases do not appear until the summer is well spent, or in the fall months. This can also be accounted for by the fact that the insect season is short in the North, and they do not appear there until after we have had them in the South for some months.

Much inoculative work has been done in trying to transmit pellagra to the lower animals with practically no success.

There is no tissue or body fluid that has not in some way been injected into the monkey; and yet they have not succumbed to the disease. We would expect such to be the case if the disease requires a separate cycle of existence in the body of an intermediate host.

So far the experiments with the stable fly have not been conclusive, but at the same time some of them have been encouraging to say the least, so encouraging as to stimulate further work along that line.

Recently the theory that the disease is due to some faulty diet has been revived, or given impetus, but to my mind there are many good arguments against that theory. If due to a faulty diet we should have seen the disease in this country long before we did and we ought to see it occur more frequently in the same family where they have the same food. Then there are many of the poorer class whose diet is very limited and yet who have so far not contracted the disease. The fact that it shows a marked improvement of itself in the fall and winter and returns in the spring when the diet has been but little changed makes it hard for me to accept the diet theory. Then there are patients who are placed on a good wholesome diet because they have the disease and who get better in the winter only to have a return of the disease in the following spring even though they have kept up the better diet containing all the essential food elements. Now it is true that those who are in a run down condition physically and nervously and have been in poor health for some time seem more susceptible, but that can be accounted for in the fact that they have less resistance, not only to pellagra, but to any other disease.

At any rate there are many workers in the field and the chance is that we will have something definite in regard to the cause before very long.

As to the course of the disease there is no time limit to it. Some cases run for several years while others run a short course and get well or die in a few weeks. The cases seen now do not seem to be quite so severe as when we first found the disease in this country. That perhaps is due to the fact that most diseases when first appearing in a community attack the weaker ones first, those who have but little resistance and who soon succumb. Accordingly, now that the weaker ones have had the disease and have been taken away, we see it in a milder and more chronic form. This, too, would account for the lower death rate.

The prognosis now is much more favorable than it was, and at least 80 per cent get well, permanently well.

We do not see diarrhea as such a prominent symptom; most likely for the same reason.

Of course, the fact that more is known about the treatment accounts in a measure for some of the above facts.

Of the symptoms we might write many pages and then not feel that the subject had been covered. There is probably not a symptom that has not been known to be the first to appear in some cases, so that we do not find the same beginning in all cases. This, of course, leads to the diagnosis not being made early in many cases. Probably a history of an indefinite stomach trouble is found in most cases. This history extending over a period of weeks, months, or even years in some cases. Along with this we frequently get a history of "nervousness" which is also of an indefinite nature. Sleeplessness also is an early and frequent symptom. The burning in the stomach or mouth or hands and feet is so characteristic, and so seldom found in other troubles that it—with the above history—is a valuable aid to the early diagnosis.

During this time the patient is usually losing weight, and this loss is so marked as to amount to from ten to one hundred or more pounds, and that sometimes without the patient's appearing to be particularly sick. There is probably no other disease in which we find such a pronounced loss of weight except tuberculosis and cancer of the stomach.

The eruption from which the disease gets its name, and without which we are loth to make the positive diagnosis, is usually rather a late symptom, and does not occur frequently until after the patient is rather far advanced, and often, too, when treatment cannot be of much avail. When it does appear there should be no difficulty in making the diagnosis, as its symmetry and peculiar appearance are characteristic, and belong to no other disease.

The sore mouth with its peculiar redness and rawness is not often seen in any other condition, unless it be in uremia or chronic alcoholism. The real sore mouth following a history like that above mentioned—and which is a common one—is enough to make one very suspicious, and is enough to justify prophylactic measures without waiting further for more symptoms.

As to the treatment: We certainly know enough about the disease to think of prophylaxis, and to begin some kind of care and treatment without waiting for all the symptoms, or waiting to be absolutely certain about the diagnosis. Many physicians today become suspicious of the disease long before they can be certain, and as we have no treatment so far that is harmful, even to the well, it seems to me wise to begin—once a suspicion is aroused. If this plan were carried out no doubt many more would recover and fewer would become confirmed invalids.

It would not matter if we could then never be certain of the diagnosis. It is the recovery of the patient we want, and if he gets well under the prophylactic treatment, or any treatment started early, we should be satisfied with having done our duty.

There are many treatments; in fact, almost all known remedies have been used in the treatment of this disease, and many have been heralded as possessing, probably, some specific value.

But we have no real specific yet. Some form of arsenic probably does best, and the form to be used differs with the physician. Personally I prefer the sodium cacodylate, which I believe does best in about five-grain doses given once daily by hypodermic. This can be kept up for months without any harmful symptoms, though of course it ought to be given under the eye of the physician, who should watch his patient for any signs of arsenic poisoning.

Another remedy that I use as a routine is dilute hydrochloric acid, which is given in doses ranging from ten to forty drops thirty minutes after each meal. The size dose depends on whether the patient is having any diarrhea. If so, the dose is increased accordingly, until the diarrhea is better, and then reduced. The reason for the acid is that the stomach of these patients is usually deficient in this normally present acid, and since it is very important in the digestion it should be supplied when not there in the normal amounts. This seems to have quite a beneficial effect on the diarrhea, and has led me to believe that not infrequently the diarrhea is due to the absence of this acid, which causes an undigested mass to be thrown into the bowel, thereby irritating it and producing the diarrhea.

Whether this be true or not, I have seen many cases of pellagra having a bad diarrhea clear up immediately after the acid was begun.

For the sore mouth I have gotten satisfactory results with a mouth wash of an alkaline nature containing potassium chlorate.

For the sore hands or the eruption it seems sufficient to keep them oiled with cold cream or some other mild oily substance.

When the diarrhea is quite bad the bismuth salts give good results and help the acid do its work of checking the bowels.

To keep the patient in bed for some weeks, or at least during the acute stage, seems to me to be very important and half the battle. No sick person does well when up and about, or attending to business, and always does better when he goes to bed and makes it a business to get well.

Now there are many symptoms that I have not mentioned, and many other things that I might have written about, but my letter would be too long and tiresome, and so I have tried to write the things that would interest you most, and to bring out the prominent points. I hear many patients say that their doctor told them that pellagra is not curable. This is not true and I doubt if the doctor said it, but he should try to state his facts to his patient so plainly that he will not be misquoted.

Yours very truly,

WILMER L. ALLISON, M. D.

---

An epidemic of spinal meningitis is raging in Georgetown, Ark. Several persons have died, four within twenty-four hours, and thirteen are in a serious condition. The town is quarantined.

---

A splendid method to mask the odor of kerosene or gasoline is to throw a lump of ammonium carbonate into the containers of the aforesaid malodorous liquids and keep it there. Try it; it works wonders.

---

The Lockhart people have planned a shower for the new hospital, which is quite a new idea. We have heard of showers for brides, and showers for babies, but this is the first time we have heard of a shower for a hospital.

---

Quite a stir was created in medical circles of Dallas when thirty-six of the forty-two physicians on the medical staff of the St. Paul's Sanitarium tendered their resignation. When asked their reasons for resigning they refused to make a statement.

---

It is claimed by one of the aldermen that the dirty windows in the street cars have caused a new disease among chicagoans. Its symptoms are tired eyes, due to the strain on peering through the darkened glass.

